# The effects of statins on dental and oral health: a review of preclinical and clinical studies

**DOI:** 10.1186/s12967-020-02326-8

**Published:** 2020-04-06

**Authors:** Shabnam Tahamtan, Farinaz Shirban, Mohammad Bagherniya, Thomas P. Johnston, Amirhossein Sahebkar

**Affiliations:** 1 Dental Research Center, Department of Orthodontics, Dental Research Institute, Isfahan University of Medical Sciences, Isfahan, Iran; 2 Department of Community Nutrition, School of Nutrition and Food Science, Food Security Research Center, Isfahan University of Medical Sciences, Isfahan, Iran; 3 Division of Pharmacology and Pharmaceutical Sciences, School of Pharmacy, University of Missouri-Kansas City, Kansas City, MO USA; 4 Halal Research Center of IRI, FDA, Tehran, Iran; 5 Neurogenic Inflammation Research Center, Mashhad University of Medical Sciences, Mashhad, Iran; 6 Biotechnology Research Center, Pharmaceutical Technology Institute, Mashhad University of Medical Sciences, Mashhad, Iran; 7 Department of Medical Biotechnology, School of Medicine, Mashhad University of Medical Sciences, P.O. Box: 91779-48564 Mashhad, Iran

**Keywords:** Statin, Periodontal disease, Dental health, Oral health, Therapeutic agent

## Abstract

The statin family of drugs are safe and effective therapeutic agents for the treatment of arteriosclerotic cardiovascular disease (CVD). Due to a wide range of health benefits in addition to their cholesterol lowering properties, statins have recently attracted significant attention as a new treatment strategy for several conditions, which are not directly related to normalizing a lipid profile and preventing CVD. Statins exert a variety of beneficial effects on different aspects of oral health, which includes their positive effects on bone metabolism, their anti-inflammatory and antioxidant properties, and their potential effects on epithelization and wound healing. Additionally, they possess antimicrobial, antiviral, and fungicidal properties, which makes this class of drugs attractive to the field of periodontal diseases and oral and dental health. However, to the best of our knowledge, there has been no comprehensive study to date, which has investigated the effects of statin drugs on different aspects of dental and oral health. Therefore, the primary objective of this paper was to review the effect of statins on dental and oral health. Results of our extensive review have indicated that statins possess remarkable and promising effects on several aspects of dental and oral health including chronic periodontitis, alveolar bone loss due to either extraction or chronic periodontitis, osseointegration of implants, dental pulp cells, orthodontic tooth movement, and orthodontic relapse, tissue healing (wound/bone healing), salivary gland function, and finally, anti-cancer effects. Hence, statins can be considered as novel, safe, inexpensive, and widely-accessible therapeutic agents to improve different aspects of dental and oral health.

## Introduction

The burden of oral diseases such as dental caries, periodontal diseases, tooth loss, lesions of the oral mucosa including those resulting from trauma, oropharyngeal cancers, and oral disease associated with human immunodeficiency virus/acquired immunodeficiency syndrome (HIV/AIDS), is very high in both developing and developed countries and is considered a major public health concern globally [1–3]. It has been recently reported that the total cost of treatment of dental diseases was $544.41 billion worldwide in 2015 [4]. One of the most prevalent conditions is periodontal disease, which affects approximately 20–50% of the global population [5]. Moreover, systemic diseases such as cardiovascular disease (CVD), diabetes, and adverse pregnancy outcomes are often associated with periodontal disease [5–9]. Therefore, incorporation of preventative strategies for oral diseases is crucial, particularly because reducing the incidence of oral and periodontal diseases can lead to a reduction in various systemic diseases. Considering the fact that general health and quality of life are profoundly affected by oral health and the very high prevalence of oral and dental diseases, finding new, effective, and safe therapeutic agents to increase oral health is definitely indispensable [5, 10–13].

The statin family of drugs represent safe and effective therapeutic agents to reduce cholesterol biosynthesis in the liver and reduction of the levels of low-density lipoprotein cholesterol (LDL-C), which are causally associated with arteriosclerotic cardiovascular disease [14–20].

**Fig. 1:**
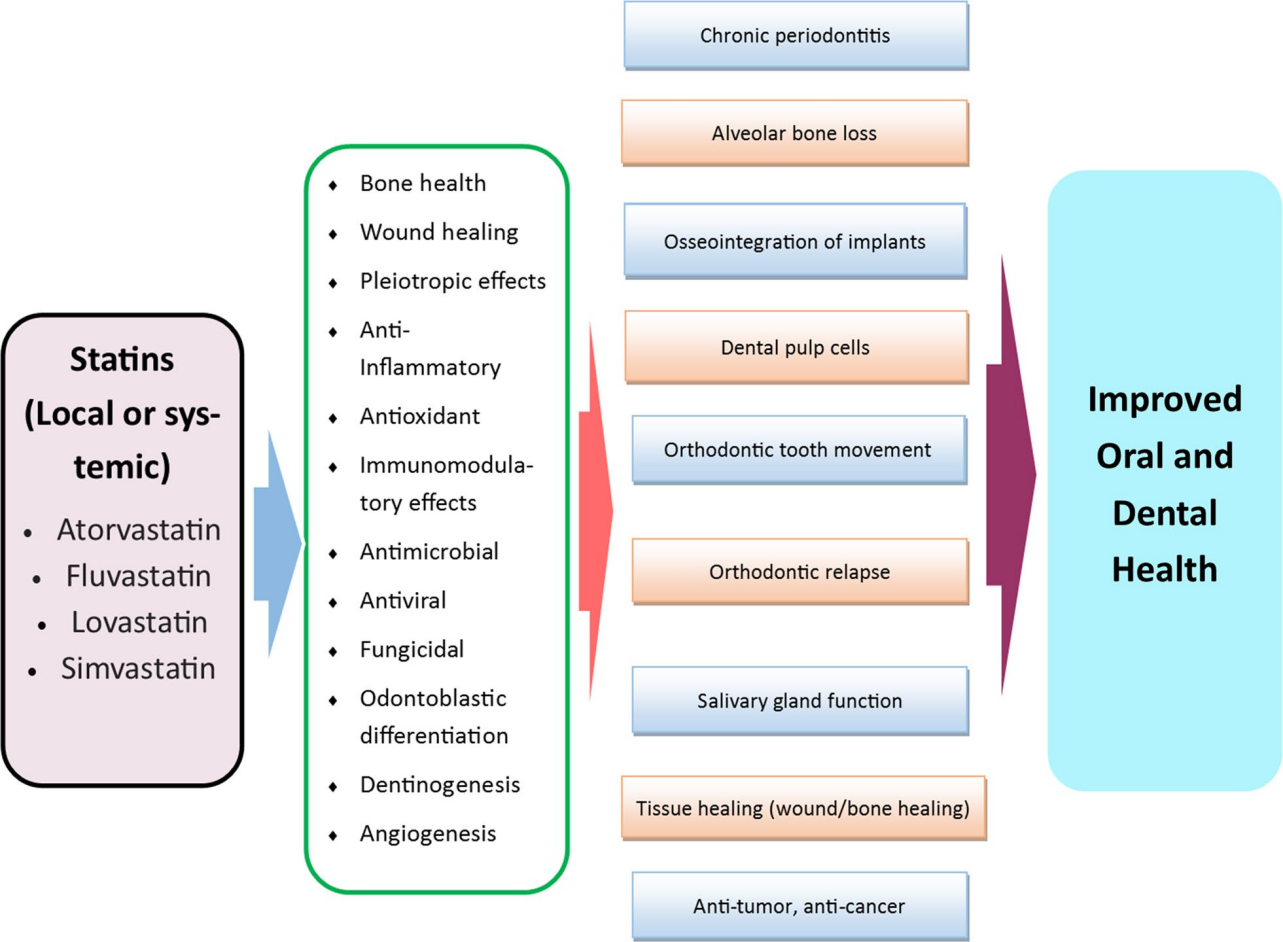
Schematic summary of pathways of the effect of Statins on different aspects of Oral and Dental health and its potential related mechanisms

Beyond their potent lipid-lowering effect, which reduces both cardiovascular risk and mortality, it is reported that statins have several promising effects on human health. These pleiotropic effects include improved endothelial function, anti-inflammatory, antioxidant properties, immunomodulatory action, and anti-thrombotic effects [21–29]. Recently, a growing body of evidence suggest that statins might have promising effects on oral and dental health through different mechanisms (Fig. 1). For example, it has been well-documented that statins exert anabolic effects on bone metabolism in different ways. Statins stimulate differentiation of osteoblastic bone marrow stem cells by increasing the gene expression of bone morphogenic protein-2 (BMP-2). They also increase bone formation by inhibiting the apoptosis of osteoblasts [30]. Bone is constantly undergoing remodeling, and it is known that statins inhibit osteoclastic bone activity during high bone turnover, which inhibits bone resorption [24, 31]. Moreover, Statin drugs also inhibit enzymes involved with the degradation of tissue [i.e., matrix metalloproteinases (MMPs)] and improve epithelization and wound healing [24, 32, 33]. Statins also exert an effect on dentin and pulp regeneration [34–36]. As it pertains to various oral cancers, statins can also inhibit the growth, invasion, metastasis, cellular proliferation and differentiation, and cell cycle regulation of tumor cells [37, 38].

Antimicrobial, antiviral, and fungicidal properties are some of the other therapeutic benefits of statins [39, 40]. The antibacterial effects of statins against certain microorganisms, including periodontal pathogens, have been reported [41, 42]. Therefore, the antimicrobial effects of statins, as well as their anti-inflammatory, immunomodulatory, anticancer, wound healing and bone-forming properties have justified their relevance in periodontology for both the prevention of alveolar bone loss and in periodontal therapy as an adjunct to scaling and root planing (SRP) [43, 44]. Furthermore, modulation of bone formation and inflammation by statins can affect orthodontic tooth movement (OTM) and orthodontic relapse [45, 46]. Bone modulation properties of Statins also had promising effects on osseointegration of implants in animal studies [47].

Considering the potential beneficial effects of statins on oral diseases, to the best of the authors’ knowledge, there is no conclusive study that summarizes the aforementioned findings of previous studies regarding the association between statins and various aspects of oral health. Thus, the primary objective of this review was to evaluate recent pre-clinical and clinical studies investigating the effect of statins on different aspects of dental and oral health. Secondary objectives associated with this review were to report the drug delivery strategies used for statins and the drug concentrations employed.

## Materials and methods

The reporting of this narrative review has been conducted in accordance with the PRISMA guidelines. A systematic electronic search of the Web of Science, Medline, SCOPUS and Google Scholar databases were using the following search terms in titles and abstracts: Periodontitis OR periodontal disease OR alveolar bone loss OR periodontal attachment loss OR periodontal pocket OR oral health OR oral diseases OR dental diseases OR oral cancer AND simvastatin OR statin OR rosuvastatin OR atorvastatin OR cerivastatin OR mevastatin OR lovastatin OR pravastatin OR Fluvastatin OR pitavastatin OR Hydroxymethylglutaryl-CoA Reductase Inhibitors. The literature was searched from January 1990 to March 2019 and only articles published in English were included. After the initial electronic search and a careful independent screening of the titles and abstracts by two researchers (FS and MB), a total of 69 papers were considered for possible inclusion. Retrieval of the 69 abstracts led to 68 full-text articles being selected. From these 69 studies, 20 studies were excluded because they were molecular in vitro studies. Studies were included if they were conducted on animal or human subjects. The methods used to ascertain the effectiveness of statins are presented for each study in the tables contained in this review. Only results that reached statistical significance have been reported (i.e., p < 0.05).

## Results

### The effect of statins on chronic periodontitis

The effectiveness of statins on chronic periodontitis has been reported in 6 systematic reviews and meta-analysis studies (Table 1). Three studies evaluated both local and systemic use of statins [32, 44, 48], while in three other studies, only local delivery of statins was investigated [43, 49, 50].

**Table 1:** The effect of Statins on dental and oral diseases based on systematic review and meta-analysis

Author, year	Type of the study	Number of included studies	Intervention/control	Local or systemic delivery of statins	Main outcomes
Bertl K et al. 2017 [32]	Systematic review and meta-analysis of periclinical in vivo trials	16 Studies in experimentally induced periodontitis (EIP) models and 7 studies in acute/chronified periodontal defect (ACP) models	Effect of local and/or systemic statin use on periodontal tissues in preclinical in vivo studies	Local and systemic use	Statin use (Simvastatin, Atorvastatin or Rosuvastatin) in periodontal indications has a positive effect on periodontal tissue parameters. Meta-analysis based on 14 EIP trials confirmed a significant benefit of local and systemic Statin use in terms of alveolar bone level/amount; meta-regression revealed that Statin type exhibited a significant effect in favor of Atorvastatin
Akram Z et al. 2017 [43]	Meta-analysis of clinical trials	11	Treatment with statins as an adjunct to scaling and root planing (SRP) in the treatment of chronic perio-dontitis (CP) compared to SRP with placebo gel	Local and systemic use	All of the studies showed that SRP and adjunctive Statin delivery was effective in the treatment of CP at follow up. In all of the included studies, periodontal inflammatory parameters (PD, CAL, and BD fill) showed significantly higher improvements in the test group (Statin delivery + SRP) compared to the control group (SRP + placebo/SRP)
Bertl K et al. 2017 [44]	Systematic review and meta-analysis of clinical trials	19	Treatment with statins as an adjunct to non-surgical and surgical periodontal therapy compared to no treatment or periodontal therapy alone	Local and systemic use	Significant additional clinical and radiographic improvements are obtained after local, but not systemic, statin use as an adjunct to SRP in deep pockets associated with intrabony defects and seemingly with furcation defects; intrasurgical statin application seems similarly beneficial. There are differences among the various statin types, in terms of efficacy, and RSV appears as the most efficacious for local application
Kellesarian SV et al. 2017 [47]	Systematic review of prospective animal trials	19	Local and systemic statin delivery to enhance the osseointegration of implants.	Local and systemic use	On experimental grounds, local and systemic statin delivery seems to enhance osseointegration
Ting M et al.2016 [51]	Systematic review of in vitro trials	26	Orally adminis- tered statins as an antimicrobial agents for microorganisms infecting oral and perioral tissues	Systemic use	Studies show that most statins exhibit antimicrobial effects against various oral microorganisms. Simvastatin is most effective against the periodontal pathogens *Aggregatibacter actinomycetemcomitans* and *Porphyromonas gingivalis*, and against most dental plaque bacteria, including *Streptococcus mutans*. Statins also exhibit antiviral properties against human cytomegalovirus, hepatitis B virus, and hepatitis C virus, and have antifungal properties against *Candida albicans*, *Aspergillus fumigatus*, and *Zygomycetes* spp.
Pavan LM et al.2015 [124]	Systematic review of in vitro or in vivo animal trials	18	Treatment with statins alone or as an adjunct to chemotherapy and/or radiotherapy approaches in the treatment of head and neck squamous cell carcinom (HNSCC) compared to no treatment or chemotherapy and/or radiotherapy approaches alone.	Systemic use	Statins have a significant effect on HNSCC cell lines with respect to cell viability, cell cycle, cell death, and the regulation of protein expression levels involved in pathways of carcinogenesis, which corroborates with the potential in vitro anti-tumor effects
De Monès et E al. 2014 [71]	A narrative review of preclinical and clinical studies	21	Treatment with statins as an agent to reduce alveolar bone resorption following extraction or periodontal disease	Local and systemic use	Statins reduced significantly alveolar bone resorption observed during periodontal disease and after tooth extraction. Oral administration was effective using high statin concentrations although local administration using a biodegradable carrier was effective with lower concentrations
Estanislau IM et al. 2015 [48]	Systematic review of clinical trials	11	Treatment with statins as an adjunct to scaling and root planing (SRP) in the treatment of chronic perio- dontitis compared to SRP with placebo.	Local and systemic use	Statins have beneficial effects, stimulating bone formation, decrease of inflammation and immunomodulation. This implies that this group of drugs might have a great potential to improve the therapeutic effect in the treatment of periodontitis, since they are safe and not costly, but not to substitute the standard of periodontal treatment, which consists in removing microorganisms
Fu JH et al. (2012) [91]	Review	7 Relevant papers, which were not reviews or in vitro studies. All animal model	Fluvastatin, Simvastatin and Rosuvastatin	Local and systemic	Evidence seems to suggest that statins may increase bone formation, which may be beneficial to dental implant healing
Moraschini V et al. 2018 [92]	Systematic review of animal studies	12	Topical and systemic statins as a pro-osteogenic agent to enhance the osseointegration of dental implants	Topical and systemic use	Findings of all of the selected studies indicated that statins (simvastatin and fluvastatin) had a statistically significant positive effect on bone formation around implants
Meza-Mauricio J et al. 2018 [49]	Systematic review and meta-analysis of clinical trials	13	Treatment with statins as adjuncts to scaling and root planing (SRP) in chronic periodontitis in comparison to the SRP alone or with placebo	Local	SRP + statin treatment (with at least 6 months of follow-up after randomization) had statistical significant effects on clinical attachment level gain, probing pocket depth reduction, modified sulcus bleeding index, and intrabony defect depth compared with SRP alone or with placebo
Muniz Fwmget al. 2018 [50]	Systematic review and meta-analysis of clinical trials	10	Treatment with statins as adjunct to mechanical periodontal therapy compared with mechanical periodontal therapy and a placebo gel	Local	Taking of simvastatin rosuvastatin, and atorvastatin as adjunct to mechanical periodontal therapy resulted in a dramatic reduction in probing pocket depth compared with mechanical periodontal therapy and a placebo gel. A significant improvement was found in resolution of intrabony defect in repose to the simvastatin and rosuvastatin use as an adjunct therapy compared with control group. Taking simvastatin as an adjunct therapy was significantly associated with clinical attachment level gain compared with control group. In all of the measured parameters, simvastatin is the only drug with additional beneficial effects

#### Statins as an adjunct to non-surgical periodontal treatment

All six studies evaluated the effect of statins as an adjunct to non-surgical periodontal treatment [scaling and root planing therapy (SRP)].

All of the studies showed that SRP + statin treatment resulted in a statistically significant improvement in periodontal parameters such as an increase in clinical attachment level (CAL), a reduction in the probing pocket depth (PD), a lower sulcus bleeding index, and a reduction in the intrabony defect depth [32, 43, 44, 48–50].

One of the studies reported that local, but not systemic statin use, caused a significantly greater gain in the clinical attachment level, less residual probing pocket depth (PD), radiographic defect depth (RDD), and reduction in the bleeding index [32].

Two studies revealed that the type of statin used was associated with periodontal outcomes. One study reported rosuvastatin as the most effective [32], while another study reported statistically significant effects with atorvastatin [44].

#### Statins as an adjunct to surgical periodontal treatment

Two studies evaluated the effectiveness of statins as an adjunct therapy to surgical treatment. These studies reported that the use of statins together with surgical treatment of intrabony defects resulted in significant improvement in clinical parameters such as residual PD and gain in CAL (Table 2) [32, 44].

**Table 2:** The effect of statins on dental and oral diseases based on clinical and preclinical studies

Author, year	Type of the study	Target tissue/cells	Sample size	Statins/intervention	Dose per day	Duration of treatment	Local or systemic delivery of statins	Main outcomes
Madi M et al. 2017 [116]	Randomized controlled trial (RCT)	Palatal donor site following free gingival graft (FGG)	40	Group I: Simvastatin suspension (S) group II: simvastatin/chitosan gel (SC) group III: chitosan gel (C) group IV: petroleum gel (P)	1 mL of the gel or the suspension (final concentration of simvastatin is 10 mg/ml) three times/day.	7 days post-operatively.	Local	Topical application of S/C gel could be used as a novel therapeutic modality that improved healing and reduced pain in the palatal donor site following FGG procedure
Noronha Oliveira M et al. 2017 [66]	RCT	Maxillary third molars postextraction sockets	13 patients(26 sockets)	1. Poly (d, l-Lactide-co-glycolide)/hydroxyapatite/*b*-TCP (PLGA/HA/*b*-TCP) with 2.0% simvas-tatin scaffold(PLGA/HA/S) 2. Deproteinized bovine bone min-eral with 10% collagen at the bone crest level (DBBM-C) 3. Poly (d, l-Lactide-co-glycolide)/hydroxyapatite/*b*-TCP (PLGA/HA/*b*-TCP) 4.No treatment group	–	3 months	Local	Poly (d, l-lactide-co-glycolide) with hydroxyapatite/b-TCP (PLGA/HA/b-TCP) scaffolds, with and without simvastatin, failed to obtain the initial expected results and presented more complications. Scaf- folds with simvastatin showed to be superior, with less clinical complications than scaffolds without simvastatin
Lin HP et al. 2017 [35]	In vitro + in vivo animal study	Target tissue (in vitro):Human dental pulp cells. In vivo animal study:The left and right first maxillary molars of male Wistar rats	36 Rats	PLGA–lovastatin nanoparticles on human dental pulp Animal study: 1. PLGA–lovastatin nanoparticles 2. ProRoot MTA 3. no treatment	100 µg/mL for dental pulp capping	2 to 4 weeks	Local	PLGA–lovastatin nanoparticles showed an effective controlled release to the 44th day. The nanoparticles exhibited good biocompatibility and superior osteogenic and odontogenic potential to dental pulp cells. They also promoted the fo mation of tubular reparative dentin and a complete dentinal bridge at the pulp exposure site in rat teeth. Therefore, this local delivery device could be used as an adjunctive treatment in direct pulp capping
Dolci GS et al. 2016 [109]	Animal study	Maxillary right molar of male wistar rats	36 Rats	1. Atorvastatin 2. Saline solution	15 mg/kg	For 7, 14, or 21 days	Systemic	Statin-induced OPG overexpression reduces relapse after orthodontic tooth movement, in a phenomenon correlated with decreased osteoclast counts. This phenomenon sheds light on OPG as a molecular target that modulates maxillary bone metabolism and orthodontic relapse
Türer A et al. 2016 [118]	Animal study	Right side of rat’s mandibular	22 rats	1. absorbable collogen sponge with saline solution containing 1 mg RSV (14 and 28 days) 2. Sterile saline treated absorbable collogen sponge(14 and 28 days)	–	2 to 4 weeks	Local	Group R-14 had significantly more new bone at 2 weeks compared with group C-14. Connective tissue volumes were also significantly higher in R-14. New bone and connective tissue volume differences were not statistically significant between groups C-28 and R-28. Locally administered RSV enhances early bone regeneration on mandibular fracture in rats
Pokhrel NK et al. 2017 [76]	In vitro and in vivo animal study	Mouse bone marrow macrophages (BMMs) animal study: male ICR mice	28 mice	Fluvastatin	3 mg/kg	On 1,4 and 7 days	Systemic	Fluvastatin significantly inhibited both RANKL- and LPS-induced osteoclast differentiation in mouse BMMs. Fluvastatin also markedly reduced the expression of osteoclast differentiation marker genes as well as fusion markers. Fluvastatin reduced the generation of reactive oxygen species (ROS) upon the addition of RANKL and LPS, suggesting an anti-oxidant role. Finally, the administration of fluvastatin in mice conspicuously reduced *P. gingivalis* LPS-induced osteoclastogenesis and alveolar bone erosion in vivo
Xu L et al. 2016 [122]	Animal study	Saliva and submandibular gland tissues of mice	96 Mice	Solvent +sham irradiation (IR) (Group I), Simvastatin +sham IR (Group II), R +solvent (Group III), and IR + Simvastatin (Group IV).	10 mg/kg three times per week	1 week prior to IR through to the end of the experiment	Systemic	IR caused a significant reduction of salivary secretion and amylase activity but elevation of malondialdehyde. SMV remitted the reduction of saliva secretion and restored salivary amylase activity. The protective benefits of SMV may be attributed to scavenging malondialde-hyde, remitting collagen deposition, and reducing and delaying the elevation of transforming growth factor 1 expression induced by radiation
Goes P et al. 2016 [73]	Animal study	Maxillary second molar of wistar rats	36 Rats	0.9% saline, Atorvastatin	(0.3, 3 or 27 mg/kg)	11 days	Systemic	ATV 27 mg/kg prevented alveolar bone loss and cemental resorption, and inflammatory cell infiltration induced by ligature. ATV showed a protecting effect in the ligature-induced periodontitis, without affecting system parameters, by inhibition of inflammatory process and by its anabolic activity on the alveolar bone
Jia W et al.2016 [34]	Animal study	The pulp tissues of the canine upper incisors immature premolars of male beagle dogs	2 beagle dogs 18 immature premolars	MTA, Absorbable gelatin sponge, cDPSCs + absorbable gelatin sponge, SMV + cDPSCs + absorbable gelatin sponge	1 mmol/L SIM for dental pulp capping	10 weeks	Local	Simvastatin stimulates cDPSCs mineralization both in vivo and in vitro. It also promotes DPSC-induced pulp and dentin regeneration after pulpotomy
Jahanbin A et al. 2016 [111]	RCT	Six anterior teeth of female orthodontic patients	16 Patients	1.2% Simvastatin gel, 0.9% sodium chloride as a placebo	0.1 ml of 1.2% SMV gel	1 day	Local	Space re-opening was reduced in patients receiving Simvastatin Moreover, GI reduction was significantly greater in Sim- vastatin group compared to the control group. Simvastatin may decrease space re-opening after orthodontic space closure in human anterior teeth
Vieira GM et al. 2015 [113]	Animal study	Maxillary first molars of wistar rats	25 adult male Wistar rats	Simvastatin solution with 0.5% carboxymethyl cellulose 0.5% carboxymethyl cellulose	5 mg/kg dose	From the 19th day until the 38th day (20 days)	Systemic	Simvastatin did not inhibit the relapse of tooth movement in rats, and there was no correlation between bone density and orthodontic relapse
Assaggaf MA et al. 2015 [135]	Animal study	Human gingival samples of patients with severe gingival overgrowth who were treated with phenytoin. Anterior maxillary and mandibular gingiva of rats	28 Rats	Control groups: physiological saline, vehicle (50% water, 41% propylene glycol, and 9% ethanol) treatment groups: Phenytoin, Phenytoin plus Lovastatin	0.65 mg/kg	12 weeks	Systemic	Phenytoin-induced gingival overgrowth in mice mimics molecular aspects of human gingival overgrowth and that lovastatin normalizes the tissue morphology and the expression of the molecular markers. Findings suggest that statins may serve to prevent or attenuate phenytoin-induced human gingival overgrowth, although specific human studies are required
MirHashemi AH et al. 2013 [112]	Animal study	First maxillary left molars of rats	Thirty-six adult male Sprague–Dawley rats	No medication, carboxymethyl cellulose (CMC), Atorvastatin in CMC	5 mg/kg	21 days	Systemic	According to the results obtained in the current study, atorvastatin appears to reduce tooth movement in rats; however its effect on osteoclasts, especially osteoclastic function, requires further investigation
Pettiette MT et al. 2013 [36]	Retrospective case–control study	Mandibular molars	90	Statin, no treatment	–	–	Systemic	The significant increase of calcification and loss of vertical height of the pulp chamber observed in mandib- ular molars in patients on statin medication indicated a possible increased odontoblastic activity. Therefore, systemic statins could be a contributing factor for pulp chamber calcification
Alghofaily M et al. 2018 [104]	Cohort study	Nonsurgical root canal treatment- Apical periodontitis	30: Statins 30: No Statins	Simvastatin, Atorvastatin, Pravastatin, Rosuvastati, Lovastatin	Regular dose (10, 20, 40, and 80 mg daily)	2 to 5 years	Systemic	A significant higher healing was observed in patients who used Statins (93%) for 2 years or greater in comparison to the patients who never took statins (70%)
Wang C et al. 2018 [125]	In vitro	Human salivary adenoid cystic carcinoma (SACC)	–	Simvastatin	1) 10, 20, 30, 40, 50 and 60 μmol/l 2) microRNA-21 inhibitor (miR-21i) + 25.37 μM simvastatin	48 h	Local	In a dose-dependent manner, simvastatin dramatically inhibited the salivary adenoid cystic carcinoma (SACC-LM) cell proliferation. Resistance of SACC-LM to simvastatin was reduced by miR-21i, which lead to SACC-LM acquisition of epithelial traits, reduction in cell migration and invasion, inhibition of growth and stimulation of apoptosis
Cai WY et al. 2018 [123]	In vitro	Human salivary adenoid cystic carcinoma cell line (SACC)	–	Simvastatin	10, 20, 30, 40 and 50 μmol/l	24 to 48 h	Local	Simvastatin therapy significantly inhibited the proliferation of SACC‑83 cells, increased the percentage of cells in early and late apoptosis, inhibited the invasiveness of SACC-83 cells and downregulated the expression of survivin in SACC‑83 cells compared with untreated cells
Dolci GS et al. 2018 [108]	Animal study	Maxilla and femur bone	24 rats	Atorvastatin	15 mg/kg	14 days	Systemic	Atorvastatin significantly reduced OTM and osteoclast counts while it did not effect on the overall bone-volume ratio compared with saline (control group). Long-term statin administration had no effect on femoral endochondral ossification
AlSwafeeri H et al. 2018 [107]	Animal study	Mandibular quadrant	10 Rabbits	Simvastatin	0.5 mg per 480 μl of solution	21 days	Local	There were no significant differences in relapse magnitude and the relapse percentage between quadrant received Simvastatin and the other quadrants. A significant reduction in the area of active bone-resorptive lacunae and a significant increase in newly formed bone area were found in respond to the local Simvastatin administration
Sezavar M et al. 2018 [68]	Clinical trail	Dental sockets	20 dental sockets	Simvastatin	20 mg	2 months	Local	A non-significant higher percentages of vital bone, amorphous, trabecular bone and lower percentages of dead bone and nonosteoblastic was observed in Simvastatin group compared with control group
Rakhmatia YD et al. 2018 [67]	Animal study	The right mandibular incisors of rats	48 male rats	Fluvastatin hydroxyapatit, hydroxyapatite plus Fluvastatin, carbonate apatite, or carbonate apatite plus Fluvastatin	0.5 mg	4 weeks	Local	Highest bone volume, trabecular thickness, trabecular separation, and bone mineral density was observed in the Carbonate apatite plus Fluvastatin group, compared with the other intervention and control groups
Medeiros C.A et al. 2010 [136]	Animal study			Atorvastatin (ATV)	ATV 1, 5 or 10 mg/kg or vehicle (saline and 5% (vol/vol) ethanol)			
Rutledge J et al. 2011 [117]	Animal study (split-mouth design)		4 dog	Simvastatin	hydroxyapatite–collagen grafts +10-mg SMV (0.5 mg/kg) was placed. One week later, three weekly	2 month (1 + 3 injection) euthanized 2 months after the final injections.	Locally injected	Locally injected SMV has the ability to induce modest amounts of new bone formation in closed injection sites over a periosteal surface. buccal bone in the dehiscence defects lacking periosteum was not augmented in the SMV group
Islam M et al.2013 [37]	In vitro and in vivo	RhoC function and HNSCC metastasis		Different concentrations of Atorvastatin		The width of the scratch was measured at 0 h and after 24 h to calculate the percentage of the gap covered by the cells in this time period		RhoC [GTP] expression is greatly reduced in Atorvastatin treated head and neck squamous cell carcinoma cell lines
Varalakshmi PR et al.2013 [106]	In vitro Extracted third molar pulp tissue	Cell proliferation, cell adherence on a dent in disc, alkaline phosphatase (ALP) activity, expression of osteogenic/odontoblastic markers, and mineralization of the human dental pulp cells on experimental cement and MTA were assesed.		Simvastatin and Atorvastatin		7 and 14 day		The results suggest that a-TCP can be used for local delivery of statin as a pulp capping material to accelerate reparative dentin formation
Goes P,et al. 2014 [74]	In vivo		78 Wistar rats: Saline group— ALD groups— ATV groups- ALD +ATV groups (low doses (ALD 0.01 mg/kg + ATV 0.3 mg/kg); high/low or low/high doses (ALD 0.25 mg/kg + ATV 0.3 mg/kg and ALD 0.01 mg/kg + ATV 27 mg/kg, respectively); or high doses (ALD 0.25 mg/kg + ATV 27 mg/kg).)	Atorvastatin (ATV) Alendronate (ALD)		The rats were killed on day 11 after ligature placement		In summary, rats subjected to periodontitis and treated with the lower-dose combination of ALD and ATV (0.01 mg/kg + 0.3 mg/kg, respectively) administered prophylactically or therapeutically, showed a reduction of periodontal inflammation and alveolar bone loss without important systemic changes
Asl Aminabadi N et al. 2012 [105]	RCT		120 Primary molar teeth:	Simvastatin	Group Ι as a control, underwent DPC with calcium hydroxide. The dental pulp in group II, III and IV were directly capped with Simvastatin-based materials at concentrations of 1, 5 and 10 μM, respectively	7.41 month	Local	Statins are not recommended as an alternative for calcium hydroxide as a DPC agent
Goes P et al. 2010 [72]	Animal study		24 male Wistar rats (± 200 g)	Atorvastatin (ATV	Oral gavage either saline or ATV (1, 3 and 9 mg/kg)	11 days	Systemic oral gavage	ATV was able to prevent alveolar bone loss seen on a ligature-induced periodontitis model
Han G et al. 2010 [110]	Animal study		Thirty-two adult male Wistar rats	Simvastatin	Simvastatin at a dose of 2.5 mg per kilogram per day for 4 weeks, and animals in the control group received 0.9% sodium chloride.	4 weeks	Systemi injections	Results indicate that simvastatin inhibits the bone-resorbing activity of osteoclasts while stimulating bone formation, probably by controlling the ratio of local osteoprotegerin to RANKL in the periodontal tissues. Therefore, it might be useful for retention
Seto H et al. 2008 [77]	In vitro & Animal study		15 rats	Simvastatin	In vitro: Different concentrations of simvastatins (1.0, 3.0 and 5.0 µM; were added to the medium and the cells were cultured for 28 d. Experiments were repeated at least three times using triplicate rat calvaria cell cultures. In vivo: Fifty microlitres of phosphate-buffered saline, alone, or containing 0.2 mg of simvastatin, was injected into the subperiosteum at the buccal area of the maxillary second molar twice a week for 70 d.	Seto H et al. 2008	In vitro & Animal study	
Moriyama et al. 2008 [93]		Animal study	60 Ten-week-old female rats	Fluvastatin. Propylene glycol alginate (PGA) was used as a carrier	Implant-only group, implant with PGA group, low-dose roup [implant‏PGA containing 3 mg of fluvastatin (FS)], medium-dose group (15 mg of FS), and high-dose group (75 mg of FS).	1 and 2 weeks	Topical	Histomorphometrical and mechanical evaluations revealed the positive effect of topically applied fluvastatin on the bone around the implant
Nassar et al. 2009 [75]	Animal study		Eighty male Holtzman rats	Simvastatin	Cyclosporine A (10 mg/kg/day), Simvastatin (20 mg/kg/day), Cyclosporine A and Simvastatin concurrently (Cyclosporine A/Simvastatin) or vehicle for 30 days.	30 days	Oral daily doses (once a day) Simvastatin	The result shows that simvastatin therapy leads to a reversal of the cyclosporine A-induced bone loss, which may be mediated by downregulation of interleukin-1b and prostaglandin E2 production
Ayukawa et al. 2004 [95]	Animal study		Ten rats	Simvastatin	10 mg/kg of simvastatin daily.	30 days	Intra peritoneally	Simvastatin increases the value of both bone contact ratio to the implant and bone density around the implant

The results of the present review show that, in general, statin treatment produces a positive effect on various parameters of periodontal tissue. These findings are supported by positive clinical results after the local application of statins during nonsurgical and surgical periodontal procedures. The rationale supporting the use of statins in treating periodontal disease is based on their antimicrobial [41, 51], anti-inflammatory [52–55], and bone-promoting properties [24, 56–58]. In particular, statins have been shown to exert antimicrobial effects against *A. actinomycetemcomitans* and *Porphyromonas gingivalis*. Both bacteria are implicated in the pathogenesis of periodontitis [41, 51]. Furthermore, statins inhibit enzymes involved with the degradation of tissue (e.g., matrix metalloproteinases) and exert a proliferative effect on mesenchymal stromal cells and endothelial progenitor cells [51, 59–61]. Statins also enhance osteoblastic differentiation and viability [62, 63], increase the expression of bone morphogenetic protein, as well as vascular endothelial growth factors, and, finally, interfere with bone resorption and the process of osteoclastogenesis [64, 65]. Due to the large variation caused by different species, defect types, evaluation methods, and reporting in these studies that utilized acute/chronified periodontal defect (ACP) models, no meta-regression was possible with these studies. Furthermore, none of the preclinical in vivo periodontitis models are able to reproduce the identical conditions to naturally-occurring periodontitis in humans. Not all available statins have been evaluated to date and, therefore, further research is needed to identify the maximum effective concentration/dose and optimal drug formulation to exploit the potential synergistic effect of statins when combined with other therapeutic agents/procedures used in treating various dental diseases.

Collectively, adjunctive use of statins appears to be effective in reducing pocket depth, clinical attachment gains, and bone defect fill in chronic periodontitis, so statins may potentially be promising therapeutic agents for periodontal regeneration.

### Effects of statins on alveolar bone loss due to either extraction or chronic periodontitis

Thirteen studies investigated the effectiveness of statins on alveolar bone. Five studies evaluated alveolar bone loss following tooth extractions [66–70], while other studies were designed to investigate inflammatory periodontitis-mediated alveolar bone loss [71–78].

### The effect of statins on alveolar socket preservation following extractions: animal studies

Three animal studies investigated the effect of statins on alveolar bone resorption following tooth extractions. In two studies, a statin was applied locally with poly-lactic-co-glycolic acid (PLGA) [69, 70], while in the third study, it was delivered with hydroxyapatite (HA) or carbonate apatite (CO) [67].

Studies in which a statin was incorporated into PLGA and subsequently applied into the socket following tooth extraction have reported significantly larger alveolar ridge height and apparent bone deposition line after 12 weeks when compared to the same clinical parameter in the control group. [69, 70].

In the third study, Micro-computed tomography (micro-CT) and histological analyses revealed the greatest bone mineral density and the highest bone volume, trabecular thickness, and trabecular separation in the carbonate apatite (CO) + statin group compared to the other groups (CO, HA, and HA + statin). [67]. These three animal studies showed that statins reduced alveolar bone resorption following tooth extractions.

#### The effect of statins on alveolar socket preservation following extractions: randomized clinical studies

Two randomized clinical trials were designed to investigate the effectiveness of statins to prevent alveolar bone resorption that can arise following a tooth extraction.

One of these trials reported a higher percentage of vital, amorphous and trabecular bone in the simvastatin group after 2 months, though it should be noted that the differences were not statistically significant [68].

In the second trial, the healing of third molar post-extraction sockets were evaluated clinically and radiographically after utilizing different ridge preservation techniques. Cone beam computed tomography (CBCT) evaluations revealed that PLGA/HA/statin and PLGA/HA groups failed to form bone after 3 months, although the biomaterial with simvastatin proved to be superior to the scaffold (biomaterial) without simvastatin and the former group had less clinical complications than the latter group [66].

These two studies failed to show beneficial effects of statins on alveolar socket preservation. Further randomized clinical trials are needed to evaluate the effect of Statins on socket preservation considering the limitations of these two trials.

Tooth extraction is an acute and brief periodontal trauma but with progressive alveolar bone resorption during the first weeks following the extraction. As the alveolar bone is easily accessible during the extraction procedure, local application of statins in the sockets would seem to represent an ideal adjunctive strategy to limit resorption of the alveolar ridge [79]. In three animal studies, the local administration of statins demonstrated a significant reduction in the resorption of alveolar bone following tooth extraction. Direct local administration of a statin has been investigated and shown to result in enhanced periodontal bioavailability of the statin due to avoidance of the hepatic first-pass effect. Of note, a one-time local administration can be achieved using a suitable carrier (e.g., a PLGA or gelatin hydrogel) to provide slow and controlled release of the statin. The in vitro release kinetics of simvastatin from either a simvastatin-PLGA scaffold, or other simvastatin-hydrogel formulations, such as those employing gelatin, has been demonstrated to be gradual and constant (zero-order kinetic) [69, 70, 78].

#### Effect of statins on inflammatory alveolar bone loss caused by periodontitis: animal studies

Six animal studies in rodents using different periodontitis models investigated the effectiveness of statins on inflammatory bone loss. Both systemic and local administration of statins was evaluated.

In one of these studies, the effect of fluvastatin on *P. gingivalis* lipopolysaccharide (LPS)-induced alveolar bone loss was examined in rats. It was shown that the administration of fluvastatin in mice reduced alveolar bone erosion and *P. gingivalis* LPS-induced osteoclastogenesis [76].

In a different animal study, the anti-resorptive effects of atorvastatin (ATV) on experimental alveolar bone loss (ABL) was evaluated in Wistar rats. In this study, the administration of ATV at a dose of 27 mg/kg prevented ABL, cemental resorption, and infiltration of inflammatory cells induced by the ligature. [73].

In another animal study, it was reported that administration of a lower dose combination of alendronate (ALD) and ATV (0.01 mg/kg + 0.3 mg/kg, respectively) reduced periodontal inflammation, ABL and secretion of matrix metalloproteinases (MMP-1, MMP-2, MMP-3, and MMP-9) in ligature-induced periodontitis in rats [74].

The effect of simvastatin on bone metabolism was also evaluated in an animal study. These investigators evaluated the effects of simvastatin on ligature-induced periodontitis in rats. Microcomputed tomography images revealed that simvastatin treatment reversed the ligature-induced alveolar bone resorption and produced a 46% increase in bone height. These findings demonstrated that simvastatin had the potential to stimulate the function of osteoblasts and further suggested that topical administration of simvastatin might be an effective strategy to recover alveolar bone loss in rats [77].

An animal study was also conducted to evaluate the efficient delivery of a simvastatin solution directly to bone defects. These investigators evaluated the release of water-insoluble simvastatin using a hydrogel, which incorporated or contained statin-micelles, in a rabbit model of tooth bone defect. It was shown that the biodegradable hydrogel comprised of gelatin was able to provide the sustained release of water-insoluble simvastatin. The hydrogel appeared to augment the simvastatin-induced bone regeneration by virtue of providing biocompatible gelatin fragments and, therefore, it was suggested that the gelatin-based hydrogel may represent an effective vehicle/platform to deliver a wide range of water-insoluble drugs [78].

Finally, one article investigated the effect of simvastatin on cyclosporine A-induced ABL, which is an important negative side-effect of cyclosporine A. Overall, treatment with simvastatin as an anti-inflammatory agent improved cyclosporine A-associated ABL. In summary, this study showed that simvastatin therapy led to a reversal of cyclosporine A-induced bone loss, which is thought to be mediated by down-regulation of interleukin-1b and prostaglandin E2 production [75].

Animal studies have reported that local statin application is effective in reducing the resorption of alveolar bone in experimentally-induced periodontitis [77, 80]. Significant reductions in periodontitis-induced alveolar bone resorption has also been reported following oral statin administration [81–87]. However, it should be noted that in five animal studies, the oral dose of the statin used (simvastatin) was tenfold greater (10–30 mg/kg/day) than the dose typically used to produce cholesterol-lowering in clinical practice (up to 1 mg/kg/day) [81, 83–86]. No significant reduction in bone resorption was reported with a lower oral dose of simvastatin (3 mg/kg/day), suggesting a dose-dependent effect with a minimum threshold dose [81]. In addition to oral administration of statins to reduce bone resorption, repeated transmucosal injection of a statin has been evaluated. The statin injection protocol involved transmucosal injections once or twice a week for 3 to 10 weeks. The statin was injected in the absence of any carrier. Although this manner of statin administration to prevent bone resorption was effective in preclinical studies, this type of protocol for statin administration would obviously not be well-suited for actual clinical practice. Thus, a single local application of a statin incorporated in a suitable biocompatible carrier has been investigated in five randomized clinical studies for the treatment of chronic periodontitis in addition to conventional scaling and root planing. In these trials, significant improvement in clinical and radiological parameters was reported in the experimental group of patients after 6 and 9 months of receiving the statin when compared with corresponding measurements for the control group. The concentration of simvastatin in the aforementioned clinical trials (1.2 µg/site with a carrier) [88–90] was far lower than the doses used in the animal studies (total doses of 1.5 mg and 4 mg) [77, 80]. The use of a carrier for the protracted and controlled local delivery of a statin may enhance the bioavailability of the statin in periodontal tissues and, therefore, allows for a much lower dose of the statin to be utilized.

This review has provided ample evidence/support for the local delivery of statins as a means to significantly reduce alveolar bone resorption following tooth extraction, as well as during periodontitis. The incorporation of a statin into a biodegradable carrier for the prolonged and controlled release of the drug after a single application may represent a more convenient mode of drug delivery for clinical use, rather than repeated injections into periodontal tissues.

### The effect of statins on osseointegration of implants

To the best of our knowledge and using our search criteria described above, we uncovered six studies that had been designed to evaluate the effectiveness of the statin family of drugs on osseointegration of implants [32, 91–95].

#### Effect of statins on osseointegration of implants: animal studies

Three recent systematic reviews, which summarized the histomorphometric outcomes of animal studies, investigated the capacity of topical and systemic statins to function as a pro-osteogenic agent to improve osseointegration of dental implants. Results of all of the selected studies indicated that statins had a statistically significant increase on the formation of bone around the implants. Statin administration increased both bone formation and density, and also enhanced new bone formation (NBF) around implants and/or bone-to-implant contact. The majority of studies reported that statin administration enhanced NBF around implants in osteoporotic rats. However, one study showed that implant surfaces coated with a Statin actually impaired osseointegration [47, 91, 92].

The effect of local application of fluvastatin on osteogenesis around titanium implants was also evaluated in two animal studies. Both studies showed that at 2 and 4 weeks, however, the bone-implant contact (BIC) and MBV were both significantly higher in the group of rats that received the 75 microg dose of fluvastatin when compared to the non-fluvastatin-treated groups (p < 0.01). Furthermore, the data showed positive correlation between the MBV and the push-in strength. These results demonstrate that locally applied fluvastatin is beneficial to bone adjacent to titanium implants and suggests that that this finding may be partially explained by calcification of the peri-implant bone [94].

Another study examined the promotion of osteogenesis around titanium implants when rats were treated with simvastatin. Results showed that thicker bone trabeculae with a mesh-like structure was abundantly observed in the medullary canal in the experimental group. Additionally, both the BCR and BD in the experimental group of rats were significantly greater than these same parameters for rats in the control group [93].

Results from ~ 95% of studies show that local and systemic statin administration is effective in enhancing osseointegration and the formation of new bone around implants. These results would appear to be strong enough to conclude that osseointegration is enhanced by local and systemic administration of statins. However, there are a number of reasons it would be difficult to replicate these experimental results in a clinical setting. First, a precise route of drug administration for statin in humans must be established. For example, in some studies, simvastatin was administered by intraperitoneal injections [95, 96], whereas, in other studies, statins were administered subcutaneously [97], orally [98], by intra-osseous injection [99], or percutaneously [100]. Secondly, the dose and frequency of statin dosing varied between the studies. This reflects the fact that in a clinical situation, the route of administration, dosage, and frequency of statin dosing was not consistent in the studies included in this review, and this needs to be further clarified. It is notable that the there was a maximum 12-week follow-up period in the experimental studies [93–101]. It remains unclear whether systemic or local administration of statins in patients receiving dental implants would increase BIC and contribute to the success and survival of dental implants for greater than 5 years.

Furthermore, it should also be mentioned that the statin doses used in the animal models were approximately tenfold greater than the daily human dose of a statin. Moreover, the route of drug administration was often percutaneous instead of by the oral route of drug administration used in humans. Further complicating factors include the fact that the tibia bone in rats is structurally different from human alveolar bone, the microbial flora in the human oral cavity is more complex than that of rat tibia, and no occlusal forces around the human implants can be reproduced in the rat tibia model. Finally, when compared to locally-delivered statins in the implant osteotomy site, the first-pass effect for orally administered statins renders approximately 10–20% of the drug available to the general circulation and, thus, might not exert a significant effect on bone remodeling around the dental implants.

Studies have shown that systemic diseases such as osteoporosis jeopardize osseointegration, which leads to a reduction in implant stability [102]. However, it is well-established that statins have a beneficial effect in the treatment of osteoporosis, which has been confirmed in vivo and in clinical practice [31, 103]. However, controversial results exist, which are associated with different factors such as type of statin, route of administration, and dosage.

To sum up, local and systemic statin delivery seems to enhance osseointegration. However, further randomized clinical trials are needed to assess the role of statins in improving osseointegration around dental implants.

### The effect of statins on dental pulp cells

Seven studies evaluated the efficacy of statins on dental pulp cells [34–36, 53, 104–106]. Six studies were conducted in humans.

#### Clinical studies

In a cohort study, 30 patients using statins during either nonsurgical root canal treatment, or retreatment, and 30 patients who did not take statins were included and followed for 2 to 5 years after treatment. At the completion of the study, a significantly higher healing of preoperative apical periodontitis (periapical index was used to determine healing) was observed in the patients who used statins for 2 years or more in comparison to the patients in the control group [104].

Uncovered in our review of the literature was also a retrospective case-controlled study that investigated the role of systemic administration of statins on odontoblastic differentiation of dental pulp stem cells. Digital bitewing radiographs of mandibular molars showed a significant reduction in the height ratio of the pulp chamber in the statin group when compared with the control group. The significant increase in calcification and the loss of vertical height of the pulp chamber observed in mandibular molars in patients taking statin medication would appear to suggest increased odontoblastic activity. Therefore, systemic statins may be a contributing factor for calcification of pulp chambers [36].

The combined effect of statin and alpha-tricalcium phosphate (alpha-TCP) on odontoblastic differentiation of human dental pulp cells (HDPCs) and its comparison with mineral trioxide aggregate (MTA) were investigated in one study. TCP-simvastatin and TCP-atorvastatin promoted odontogenic differentiation in HDPCs as documented by the expression and activity of osteogenic/odontogenic markers (i.e., DSPP, DMP 1, ALP, BMP-2, and OCN), as well as the formation of mineralized nodules, which was comparable with the MTA group. The results suggested that alpha-TCP may be used for the local delivery of simvastatin as a pulp capping material to hasten the formation of reparative dentin. Moreover, as mentioned previously in this review, statins have been shown to exert anti-inflammatory effects, so this beneficial property may also help to restore inflamed pulp tissue [106].

In a different study, a randomized clinical trial evaluated the efficacy of simvastatin at concentrations of 1, 5, and 10 μM and compared to calcium hydroxide with regard to direct pulp capping of human primary molars. The results indicated that hard tissue formation and healing without inflammation occurred following the statin treatment, but at a lower rate than the calcium hydroxide [105].

Another study assessed the effect of simvastatin on tumor necrosis factor alpha (TNF-alpha)-induced synthesis of Cyr61 and C–C motif chemokine ligand-2 (CCL2) in MG-63 human osteoblasts. Cysteine-rich 61 (Cyr61) and CCL2 are potential osteolytic mediators in inflammatory bone diseases. A Western blot analysis showed that TNF-alpha stimulated Cyr61 synthesis in MG-63 cells. However, the addition of simvastatin attenuated this effect in a dose-dependent fashion. Simvastatin also reduced the levels of TNF-alpha–induced CCL2, although the inhibitory effects were restored by exogenous Cyr61. The administration of simvastatin markedly diminished the severity of experimentally-induced rat periapical lesions as determined using radiography and histopathology. There was also a decrease in the numbers of Cyr61-synthesizing osteoblasts and CD-68–positive macrophages. It has been suggested that simvastatin limits the progression of apical periodontitis, possibly by diminishing Cyr61 expression in osteoblasts and, subsequently, by reducing macrophage chemotaxis into the lesions [53].

One study was designed to evaluate the application of poly (d, l-lactide-co-glycolide acid) (PLGA) nanoparticles that contained lovastatin in direct pulp capping. The results showed less toxicity to cultured human dental pulp cells with the PLGA-lovastatin nanoparticles than free lovastatin after 5, 9, and 13 days. Additionally, PLGA-lovastatin nanoparticles induced the greatest expression of mRNA and dentin sialophosphoprotein (DSPP), dentin matrix acidic phosphoprotein 1 (DMP1), and osteocalcin (OCN) in the cultured pulp cells.

#### Animal studies

One study was designed to compare PLGA-lovastatin nanoparticles and mineral trioxide aggregate (MTA) as direct pulp capping materials for teeth using Wistar rats. Upon histological evaluation, it was revealed that MTA was superior to PLGA-lovastatin nanoparticles in terms of stimulating the formation of tubular dentin after 2 weeks. However, following an observation period of 4 weeks, it was evident that PLGA-lovastatin nanoparticles and MTA were very similar in mediating the formation of tubular reparative dentin and a complete dentinal bridge [35].

An animal study investigated the effect of different concentrations of simvastatin on the proliferation and differentiation of dental pulp stem cells (DPSCs) and pulp regeneration after pulpotomy. The results showed that simvastatin (1 mmol/L) suppressed the proliferation of canine dental pulp stem cells (cDPSCs), but the activity of alkaline phosphatase and mineral nodule formation were both significantly increased. In addition, the areas of pulp and dentin regeneration in the simvastatin group were significantly higher than these same parameters in the other groups [34].

Collectively, the results of these various studies indicate that introduction of statin-treated DPSCs into the pulp chamber can lead to coronal pulp regeneration, as well as dentin restoration. Therefore, it was concluded that statins stimulate both the mineralization of DPSCs and the formation of dentin in vivo and in vitro. It should be emphasized that statin-treated DPSCs showed an increase in the activity of ALPase and mineral nodule formation.

Statins have been investigated as a novel, alternative agent for pulp capping. Because statins exert anti-inflammatory properties as one of their many pleiotropic effects, they are of interest to practicing dentists and specialists alike. Pulp and periapical symptoms can be modulated with the use of statins, and they can also potentially decrease the development of apical periodontitis.

### Statins and orthodontic tooth movement/orthodontic relapse

A potential clinical concern during orthodontic treatment is that statin therapy may be associated with the promotion of osteogenesis and the suppression of bone resorption. Seven studies included in this review were designed to investigate the effect of statins on orthodontic tooth movement and relapse [107–113]. Only one of these studies was conducted on humans [111].

#### Effect of statins on orthodontic tooth movement/orthodontic relapse: animal studies

The effect of atorvastatin on orthodontic tooth movement (OTM) and the potential adverse effects of atorvastatin on long-bone turnover and endochondral ossification were examined in an animal study. Results indicated that atorvastatin significantly reduced OTM and osteoclast counts, while it did not affect the overall bone:volume ratio. Long-term statin administration had no effect on femoral endochondral ossification [108].

Another animal study also evaluated the effect of atorvastatin on OTM in rats. Results showed that there was a statistically significant (p < 0.05) reduction of OTM following administration of atorvastatin, but there was no significant difference (p > 0.05) in various histologic indices [root resorption, periodontal ligament (PDL) width, and osteoclast number] among the three groups [112].

The effect of local administration of simvastatin on post-treatment relapse was evaluated on 10 rabbits [114]. At post-intervention, the magnitude of relapse, as well as the percent relapse, between the two quadrants did not demonstrate differences that were statistically significant, although, based on histomorphometric analyses, a significant reduction in the area of active bone-resorptive lacunae and a significant increase in newly-formed bone area was determined in response to local simvastatin administration [107].

Another animal study evaluated the effect of simvastatin on relapse of tooth movement in rats using micro-computed tomography (micro CT), and also determined whether there was a positive correlation between bone density and orthodontic relapse. Relapse and bone mineral density (BMD) were lower in the experimental group, but did not reach statistical significance. Additionally, these investigators found no correlation between bone density and orthodontic relapse [113].

The effects of simvastatin on orthodontic relapse and the remodeling of periodontal tissue following experimental tooth movement in rats was evaluated in a different animal study. Osteoprotegerin was strongly expressed on both sides of the PDL in the simvastatin group when compared to the control group. All results demonstrated that simvastatin effectively stimulated bone formation. This suggests that simvastatin has the potential to accelerate tooth stability in a new position, assist retention of teeth, and stabilize any loosened teeth in patients with periodontal disease. Furthermore, RANKL was strongly expressed, and its expression on both sides of the PDL was higher in the control group than in the simvastatin group [110].

The effect of Atorvastatin (ATV) on orthodontic relapse and osteoclastogenesis was investigated in one animal study by evaluating the expression of RANKL and osteoprotegerin (OPG). The results showed that ATV decreased tooth relapse and osteoclast counts which were positively correlated. Moreover, there was an increase in periodontal expression of OPG, but not RANKL with statin administration [109].

#### The effect of Statins on orthodontic tooth movement/orthodontic relapse: clinical trial

The effect of simvastatin on space re-opening after orthodontic space closure and its impact on the gingival index (GI) and clinical attachment loss (CAL) were evaluated in a randomized clinical trial. Those patients that had received the simvastatin gel had a significant reduction in space re-opening (p < 0.001). Moreover, GI reduction was significantly greater (p < 0.001) in the simvastatin group when compared to the control group. However, there was no significant difference between the experimental and control groups with regard to CAL [111].

In general, it has been shown in numerous studies that statins reduce orthodontic relapse. Findings in the above studies suggest that daily administration of statins affects bone resorption during orthodontic relapse, which was demonstrated by a significant decrease in the overall osteoclast count. Taken together, these results demonstrate that statins mediate the inhibition of osteoclastogenesis and may represent a potential target with which to minimize orthodontic relapse.

In addition, there have been some studies that have reported that simvastatin (SMV) promotes bone formation, as well as inhibits osteoclast activity [65, 115]. Statins can stimulate regeneration of new bone in rodents, and this effect appears to be related to increased expression of bone morphogenic protein-2 in osteogenic cells. Simvastatin at low concentrations exhibits a positive effect on the reproduction and differentiation of PDL cells to osteoblasts. SMV also limits osteoclast activity, while stimulating the formation of bone. The effects of SMV on osteoclast activity and bone formation would seem to suggest that its action is exerted by regulating the ratio of local osteoprotegerin to RANKL in periodontal tissue. Therefore, it has been suggested that SMV might be useful for tooth retention [110].

As mentioned above, the use of statins to inhibit osteoclastogenesis may represent a viable therapeutic strategy with which to minimize orthodontic relapse.

### Statins and tissue healing (wound/bone healing)

Three studies evaluated the efficacy of statins on wound and bone healing [116–118].

#### The effect of Statins on tissue healing: animal studies

One study using rats investigated the effect of local administration of rosuvastatin (RSV) on mandibular fracture healing. Animals in groups with the number 14 or 28 were euthanized 14 days, or 28 days, after the operation, respectively. Stereologic analysis showed that the rats contained in group R-14 had significantly more new bone at 2 weeks compared with those rats in group C-14. The volume of connective tissue was also significantly greater in rats included in group R-14, although there was no significant difference in this parameter between groups C-28 and R-28. These authors concluded that locally-administered RSV enhances early bone regeneration after mandibular fracture in rats [118].

The effectiveness of simvastatin injections in an ethanol carrier was evaluated in an animal study (beagle dogs) using several human-like clinical situations (dehiscence defect on root, thin bone on root, and ridge augmentation). Application of simvastatin to alveolar bone surfaces is a clinically safe procedure with few adverse effects other than a moderate degree of swelling at the site of injection. However, the use of simvastatin did not enhance the bone width gain obtained with buccal overbuilding procedures performed with the hydroxyapatite graft and membrane in this model. Specifically, multiple injections of simvastatin did not enhance new bone deposition over dehiscence bone defects where no periosteum exists [117]. Although repeated injections of simvastatin induced new bone deposition over thin alveolar bone covering root surfaces and in edentulous sites, nevertheless, it would seem that additional prospective studies are warranted to unequivocally identify the best animal model to evaluate the effects of repeated simvastatin injections in order to prevent the loss of thin alveolar bone (dehiscence defects, recession, and post extraction bone loss).

#### The effect of statins on tissue healing: clinical studies

In one study, the effect of intra-oral topical application of simvastatin/chitosan gel (10 mg/mL) over the palatal donor site following a free gingival graft (FGG) procedure was evaluated. Statistically significant reductions in wound-healing scores were observed after 3 and 7 days for (simvastatin/chitosan gel) group when compared to other groups. A significant reduction was also observed in the visual analog scale (VAS) score on days 1, 3, 5 and 7 when compared to the other groups on the same days [116].

Statins can also accelerate epithelization and the rate of wound closure by inhibiting adhesion and extravasation of leukocytes into the site of inflammation, which can result in reduced co-stimulation of T-cells and a reduction in inflammatory cytokines. These processes both facilitate wound healing during the early stages of wound repair. Additionally, statins have been shown to enhance the infiltration of macrophages, which, in turn, stimulates the proliferation of fibroblasts, keratinocytes, and endothelial cells [119]. Stimulation of angiogenesis, which promotes infiltration of macrophages, as well as induces both the production of vascular endothelial growth factor (VEGF) and re-epithelialization, have also been reported following statin use [119–121].

In summary, studies would appear to support the premise that topical application of simvastatin is safe at a low concentration (10 mg/mL) and promotes wound healing. Together with their antibacterial activity and capacity to modulate the inflammatory process, this wound care strategy involving statins could potentially minimize the risk of bacterial infection during the wound healing process.

It can be concluded that topical application of statins represents a safe and promising treatment modality for promoting wound healing, however, additional clinical trials are needed to confirm these results.

### Statins and salivary gland function

The radioprotective potential of simvastatin (SMV) has been investigated in a murine model of radiation-induced salivary gland dysfunction in mice. The systemic administration of SMV by intraperitoneal(i.p). injection lessened the reduction in saliva secretion and restored the activity of salivary amylase [122].

Results demonstrated the potential of SMV to function as a radio-protective agent for salivary glands. The protective benefits of SMV were thought to be due to scavenging of radiation-induced free radicals, minimizing collagen deposition, and reducing/delaying the elevation of transforming growth factor β1 expression, which is induced by radiation. Therefore, it is suggested that statins may potentially represent a clinically useful treatment to alleviate the side effects of radiotherapy on salivary glands.

### Anti-tumor and anti-cancer effects of statins

Four studies have evaluated the anti-cancer effects of statins in the head and neck area [37, 123–125].

One study assessed the effect of simvastatin in combination with microRNA-21 inhibitor (miR-21i) in lung metastatic salivary adenoid cystic carcinoma (SACC-LM) cells. It should be noted that one of the most prevalent malignancies of the salivary glands is salivary adenoid cystic carcinoma (SACC). Additionally, it should be emphasized that microRNA-21 (miR-21) has a substantial effect on tumor development. Results indicated that simvastatin dramatically inhibited SACC-LM cell proliferation in a dose-dependent fashion. In addition, resistance of SACC-LM cells to simvastatin was reduced by the miR-21i, which led to an acquisition of epithelial traits, a reduction in cell migration and invasion, an inhibition of growth and, lastly, stimulation of apoptosis, in the SACC-LM cells [125].

In another recent study, the effect of simvastatin on the proliferation, invasion, and apoptosis of human SACC was investigated. Results demonstrated that exposure of SACC cells to simvastatin (10 to 50 µM) for 24 to 48 h considerably, and in a dose- and time-dependent manner, inhibited the proliferation of the SACC cells when compared to non-simvastatin treated cells. Furthermore, in response to the simvastatin, the percentage of cells in the ‘early’ and ‘late’ stages of apoptosis was increased. Finally, the invasiveness of SACC cells when exposed to simvastatin was inhibited in a dose-dependent manner, and simvastatin exposure also mediated a down-regulation in the expression of survivin (survivin is overexpressed in some types of cancers) [123].

The anti-tumor effects of statins on head and neck squamous cell carcinoma (HNSCC) were evaluated in a systematic review. Only fourteen in vitro studies that discussed the effect of statins on HNSCC were included in this review. These studies demonstrated that statins had a significant effect on HNSCC cell lines by influencing cell viability, the cell cycle, cell death, and protein expression levels that are involved in pathways associated with carcinogenesis [124].

In vitro and in vivo studies were conducted to evaluate the ability of atorvastatin to inhibit RhoC function and HNSCC metastasis. The results showed that treatment of HNSCC cell lines with atorvastatin decreased cell invasion and migration. Statin treatment also decreased the membrane fraction of Ras homolog gene family member C (RhoC) and limited the activation of two critical signaling pathways; namely, the extracellular signal-regulated kinase 1/2 (ERK1/2) and the signal transducer and activator of transcription 3 (STAT3) signaling cascades. Most importantly, in an in vivo animal model, the inhibition of RhoC resulted in a decrease in metastases when compared to placebo-treated animals. In summary, this work serves to confirm the use of statins as an adjunct treatment modality to standard therapies currently available for HNSCC [37].

Currently, new therapies are emerging for oral cancer. Advanced tumors in the recurrent stage, as well as distant metastases, are currently treated with combination therapy involving surgical resection, which is followed by radiotherapy and, more often than not, chemotherapy. Almost all cancer treatments that involve radiation or pharmacotherapy are cytotoxic and cause many adverse effects for the patient. Therefore, therapeutic approaches that are less cytotoxic are desperately needed, especially for the treatment of HNSCC [126].

It has been previously recognized that statins have anticancer effects. Some results have shown that statin monotherapy can kill more than 50% of a tumor cell burden, as well as inhibit their proliferation in a dose-dependent manner. The effect of statins, when combined with either radiation, or other chemotherapeutic agents (cisplatin, 5-fluorouracil, paclitaxel, carboplatin, and oxaplatin), on HNSCC cell viability has also been investigated. However, disappointingly, the inclusion of statins with the chemotherapeutic agents mentioned directly above had no significant effect on HNSCC cell kill [127, 128]. However, when a combination of gefitinib and lovastatin was tested using squamous cell carcinoma 9 (SCC9) cells, cell death was increased by more than 90% when compared to monotherapy with either drug [38, 129]. Similar results were reported by Dayekh et al. when they used lovastatin combined with monensin (a polyether antibiotic isolated from Streptomyces cinnamonensis) and erlotinib [130]. Using FaDu cells in in vitro clonogenic assays, de Llobet et al. showed that simvastatin enhanced the effects of radiation treatment alone and when in combination with cetuximab in terms of cell proliferation. These authors subsequently observed that the in vitro results were reflected in xenoimplanted tumors growing into subcutaneous tissue of athymic mice in which concomitant treatment with simvastatin decreased tumor growth, induced apoptosis, and increased wound healing [131].

The final products of the mevalonate pathway, which includes de novo cholesterol and isoprenoids, have a potential effect on the viability of malignant cells [132]. Several of the products from the mevalonate pathway affect cell proliferation and are necessary for various key cellular functions including maintenance of cell membrane integrity, signaling, protein synthesis, and cell cycle progression. It is thought that interruption of these processes in malignant cells can inhibit tumor growth and metastasis [133]. Simvastatin has been shown to regulate the expression of phosphorylated forms of ERK1\2 (extracellular-signal-regulated kinases) and the expression of cell cycle regulators, such as p21 and p27, in HNSCC cells [132, 134]. Additionally, atorvastatin treatment in vitro significantly decreases the active form of RhoC in HNSCC cells. Importantly, atorvastatin also mediates a significant reduction in the phosphorylated forms of ERK1/2 and STAT3, and reduces HNSCC cell motility, invasion, proliferation, and colony formation. These in vitro results provide support for the suggestion that statin exposure/administration may potentially represent a therapeutic intervention for the treatment of HNSCC [124].

Collectively, statins have significant anti-tumor effects on tumor cell lines with respect to the cell cycle, cell death, and through the regulation of protein expression involved in various carcinogenic pathways.

### The effect of statins on gingival tissue

#### The effect of statins on gingival tissue: animal studies

Gingival overgrowth has been shown to be caused by the anti-seizure medication phenytoin, calcium channel blockers, and cyclosporin. In one particular study, the efficacy of lovastatin to prevent phenytoin-induced gingival overgrowth was evaluated in mice. Lovastatin was selected based on previous analysis of tissue-specific regulation of CCN2 production in human gingival fibroblasts and because CCN2 is known to promote fibrosis and epithelial-to-mesenchymal cell transition. Lovastatin not only decreased epithelial gingival tissue growth in phenytoin-treated mice, but also altered the expression of biomarkers that indicate epithelial-to-mesenchymal cell transition. Data indicated that gingival overgrowth induced by phenytoin in mice replicates various molecular aspects of gingival overgrowth in humans. Moreover, these same authors showed that lovastatin normalized tissue morphology, a well as the expression of molecular markers evaluated in this study. Findings suggested that statins may prevent or attenuate phenytoin-induced human gingival overgrowth, although specific human studies are required to confirm this finding [135].

Cancer patients treated with cancer chemotherapeutic agents often suffer from oral mucositis (OM). The effect of atorvastatin (ATV) on OM induced by the administration of 5-fluorouracil (5-FU) to hamsters was previously investigated. OM was induced in the hamsters by repetitive intraperitoneal injections of 5-FU. ATV was effective at reducing mucosal damage and inflammation, as well as decreasing the levels of cytokines, nitrite, and myeloperoxidase activity on the 5th and 10th day of the 5-FU-induced OM. However, the highest dose of ATV (10 mg/kg) with 5-FU treatment induced hepatotoxicity and amplified leukopenia [136].

Three types of drugs induce gingival overgrowth with fibrosis and inflammation. As mentioned above, these drugs are phenytoin, cyclosporin, and nifedipine. Although these three medications are of different drug classes, they all can cause drug-induced gingival overgrowth with manifests as enlarged gingival epithelium and connective tissues that exhibits varying degrees of fibrosis and inflammation.

Phenytoin-induced gingival overgrowth is the most fibrotic form of gingival overgrowth and is associated with increased expression of CCN2, but a reduced presence of inflammatory cells. It has been shown that TGF-beta 1 is highly expressed in phenytoin-induced human gingival tissue [137, 138]. Results in mice have demonstrated that higher expression of TGF-beta 1 in the phenytoin-treated mouse group was attenuated in the presence of lovastatin. This is related to the fact that statins, which are HMG-CoA reductase inhibitors, inhibit TGF-beta 1 expression in kidney and tooth extraction sockets [135].

### Adverse effects of statins as it pertains to oral health

One study pertaining to statins and various oral health parameters was identified in this review. The article evaluated the side effects of statins in the oral cavity and characterized the symptoms after interruption of statin treatment.

Patients, aged 50–70 years old, who had been previously diagnosed with hypercholesterolemia and were receiving statin treatment were referred to a dentist’s office. Anamnesis regarding their oral symptoms was obtained on their initial visit. Statin treatment was then discontinued, which was followed by various laboratory tests and repeat visits to the dentist at seven and 15 days after statin discontinuation. Results showed that a high percentage of oral symptoms included dry mouth, itchiness, bitterness, and cough during statin therapy. There was a marked improvement in their symptoms after temporary interruption of statin treatment. This study included a relatively small number of patients and a more thorough and detailed design of experimental treatments is needed to establish a true correlation between statin treatment and oral symptoms [139].

## Conclusion

The studies reviewed in the present manuscript, while being heterogeneous in terms of the type of studies, interventional approaches, and the duration and intensity of intervention, overwhelmingly suggest that the statin family of drugs have unique beneficial effects on dental and oral health. Almost all of the studies, using different methodological approaches including observational, in vivo, in vitro, animal, and randomized clinical studies, showed that systemic and, in particular, local application of statins, plays a salient role in improving dental and oral health (Table 2).

**Fig. 2:**
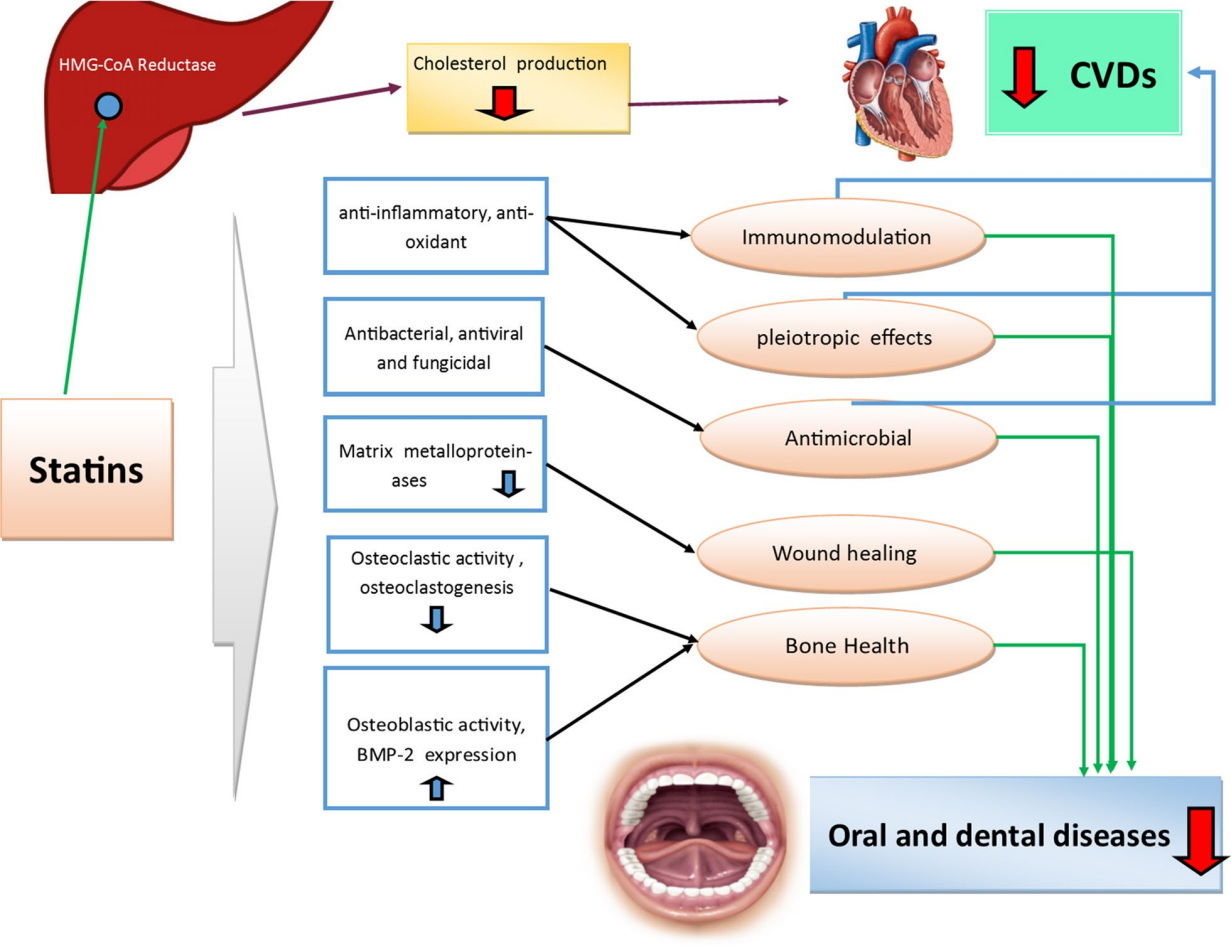
Schematic summary of pathways of the effect of Statins on Cardiovascular diseases (CVDs) and on Oral and Dental diseases and their potential related mechanisms

Based on the results of this comprehensive review, it is suggested that statins possess a remarkable beneficial effect on chronic periodontitis, alveolar bone loss, osseointegration of implants, dental pulp cells, orthodontic tooth movement and subsequent relapse, tissue healing (wound/bone healing), and salivary gland function, as well as exhibiting anti-cancer properties in the oral cavity (Fig. 2). The findings of systematic reviews and meta-analyses contained in the current review confirm the favorable effects of statins on different aspects of dental and oral health (Table 1). Presently, there are several clinical trials underway to assess the therapeutic effects of statins on dental and overall oral health, especially as it relates to periodontitis (Table 3). In the future, it is strongly suggested that larger clinical trials be conducted to assess the pleiotropic effects of statins on dental and oral health, focusing on determination of the ideal duration, dose, and specific statin (atorvastatin, fluvastatin, lovastatin, rosuvastatin, or simvastatin) for the treatment of each particular dental and/or oral condition. However, in the meantime, and based on the findings contained in the present review, it is probably safe to suggest that local, or even systemic use of statins, should be considered as a novel, safe, inexpensive, and very accessible therapeutic agent with which to improve various aspects of overall dental and oral health.

**Table 3:** List of clinical trials evaluating the effects of statins on dental and oral health (retrieved from http://www.clinicaltrials.gov)

	Drugsstatins	NCT identifier	Condition or diseases	Inclusion criteria	Exclusion criteria	Dose	Duration	Local/Systemic	Primary outcome	Phase
1	Curcumin-Simvastatin-EDTA	NCT04044417	Periodontitis	Patient suffering at least a single posterior 2–3 wall periodontal Pocket of depth ≥ 5 mm, attachment loss ≥ 3 mm, intrabony component ≥ 3 mm, plaque index ≤ 1 (Sillness and Loe, 1964) and sulcus bleeding index ≤ 1 (Mühlemann & Son, 1971), All these criteria were determined after phase I conventional periodontal therapy, Systemically healthy as evidenced by burket’s oral medicine health history question questionnaire (Glick et al. 2008)	SmokingPatient unwilling to comply with periodontal hygienic instructions. Patients under any long-term chronic medication. Vulnerable individuals	Curcumin-simvastatin paste (2% curcumin and 1.2% simvastatin)	6-months	Local	Probing depth (PD)	Phase 4 (Completed)
2	Atorvastatin, Simvastatin	NCT03745300	Chronic Periodontitis Wth Diabetes Mellitus	Well controlled type 2 diabetes mellitus patients classified based on criteria given by American diabetes association with CP with PD ≥ 5 mm and CAL ≥ 3 mm and presence of IBD ≥ 3 mm (distance between alveolar crest and base of the defect on an intraoral periapical radiograph [IOPAR] after phase I therapy	1) Patients with known allergy to statins; 2) Patients with systemic conditions/medications known to affect the periodontal status; 3) aggressive and refractory periodontitis 4) Hematological disorders 5) pregnancy/lactation; and 6) Smoking and tobacco use in any form 7) Immunocompromised individuals. 8). patients with poor plaque control. 9). non vital teeth, carious teeth warranting restorations, third molars and mobility of at least grade II were excluded	Scaling and root planing (SRP) followed by 1.2% atorvastatin or 1.2% simvastatin gel local drug delivery	6 and 9 months	Local	Change in defect depth	Phase 2 and 3 (completed)
3	Simvastatin-Methylcellulose	NCT03452891	Periodontal Diseases	Age 40-85 years, diagnosis of chronic advanced adult periodontitis, one quadrant with at least one 6-9 mm interproximal pocket, overall good systemic health, willingness to sign consent form	Systemic diseases which significantly impact periodontal inflammation and bone turnover (e.g. rheumatoid arthritis), taking drugs which significantly impact periodontal inflammation and bone turnover (e.g. chronic use of steroids or non-steroidal anti-inflammatory drug (> 325 mg/d), estrogens, bisphosphonates, calcitonin, methotrexate), surgical periodontal therapy within the past year, pregnant or breast-feeding females	Simvastatin-Methylcellulose gel will be placed in a deep (6-9 mm) periodontal pocket	6 and 12 months	Local	Change in Clinical Attachment Level (CAL)	Phase 4 (recruiting)
4	Rosuvastatin	NCT03677297	Adult Periodontitis	Age group between 30–50 years, probing pocket depth ≥ 5 mm following initial therapy, suitable interproximal angular infrabony defects of ≥ 3 mm, involved teeth should be vital and asymptomatic, systemically healthy patients, Patients who demonstrate acceptable oral hygiene prior to access flap surgery.	Systemic statin therapy, known or suspected allergy to the statin group, allergy to sulfur containing drugs, history of aggressive periodontitis, presence of gingival recession at the surgical site, mobility of study teeth ≥ grade I, use of tobacco in any form, pregnant and lactating women, patients who have received any anti-inflammatory drugs and antibiotics in the previous 6 months, H/O osteoporosis.	1.2% Rosuvastatin Gel. Insertion in infrabony defects once	6 months	Local	Bone fill	Phase 4 (completed)
5	Simvastatin	NCT03419429	Chronic Periodontitis	Patients were all healthy and free from any systemic disease, No history of antibiotic therapy or periodontal treatment for at least six months preceding the study, Patients were willing and able to return for multiple follow up visits, Periodontal defects with Probing depth > 5 mm, Clinical attachment loss > 4 mm, Standardized radiographic evidence of interproximal intrabony defect using periapical radiograph, Good level of oral hygiene (plaque and gingival indices score after initial phase therapy should be less than one)	Pregnancy, lactation for female patients, smokers, alcoholics and those receiving any medication that could affect healing of soft tissue and bone as steroids and cyclosporines, history of allergic reaction to the medications used, vulnerable groups and handicapped	Open flap procedure, 1.2%simvastatin gel applied and covering the defect with resorbable collagen occlusive membrane	9 months	Local	Probing pocket depth	Phase 4 (Completed)
6	SRP plus Rosuvastatin gel	NCT03043196	Chronic Periodontitis	Systemically healthy CP subjects aged between 30 and 50 years who are current smokers with no history of periodontal therapy or use of antibiotics in past 6 months, having sites with intrabony defect depth (IBD) ≥ 3 mm (distance between alveolar crest and base of the defect on intraoral periapical radiograph) along with PPD ≥ 5 mm or CAL ≥ 3 mm in an asymptomatic tooth were included in the study, a subject was considered as a current smoker if he regularly smoked more than 10 cigarettes/day for a minimum of 5 years	Former smokers, i.e. subjects who had previously been smokers but stopped their habit, and non-smokers were excluded. Subjects allergic to statins, on systemic statin therapy, with any known systemic disease or any other systemic inflammation/infection which could alter the course of periodontal disease and users of tobacco in any other form than cigarettes were excluded from the study	Oral prophylaxis followed by 1.2% Rosuvastatin drug in gel form placed in intrabony defects	9 months	Local	Defect depth reduction	Phase 2 and 3 (completed)
7	Lovastatin gel	NCT03178526	Alveolar Bone Loss, Chronic Periodontitis	Patients with periodontitis chronic and TEMPhas a 2 molar symmetrically in the lower jaw pockets of periodontal depth of at least 4 mm and CAL greater than 3 mm in at least one of the surfaces of the teeth, the X-ray bars from both sides of the molar regions jaw bottom, from CEJ and alveolar crest was more than 3 mm	Systemic disease, pregnancy or breastfeeding, allergy drug used, smoking, medication, not willing to consent to participate in the study, Trismus 8-The type of disease periodontal (Aggressive) 9. History of periodontal treatment in the previous 6 months 10-orthodontic treatment	Lovastatin gel1.2% topical gel was put into teh periodontal pocket using an insulin syringe	3 months	Local	Bone defect fill	Phase 2 and 3 (completed)

## Data Availability

Not applicable.

## Abbreviations

HIV/AIDS: Human immunodeficiency virus/acquired immunodeficiency syndrome
NHANES: National Health and Nutrition Examination Survey
CVD: Cardiovascular disease
HMG-CoA: 3-hydroxy-3-methylglutaryl reductase A
LDL-C: Low-density lipoprotein cholesterol
BMP-2: Bone morphogenic protein-2
RANK: Receptor activator of nuclear kappa B
RANKL: Receptor activator of nuclear kappa B ligand
OPG: Osteoprotegerin
CRP: C-reactive protein
MMPs: Matrix metalloproteinases
SRP: Scaling and root planning
OTM: Orthodontic tooth movement
CAL: Clinical attachment level
PD: Pocket depth
RDD: Radiographic defect depth
ACP: Acute/chronified periodontal defects
PLGA: Poly-lactic-co-glycolic acid
HA: Hydroxyapatite
CO: Carbonate apatite
TGF-B1: Transforming growth factor B1
VEGF: Vascular endothelial growth factor
Micro-CT: Micro-computed tomography
CBCT: Cone beam computed tomography
LPS: Lipopolysaccharide
ATV: Atorvastatin
ABL: Alveolar bone loss
ALD: Alendronate
NBF: New bone formation
PGA: Propylene glycol alginate
MBV: Mineralized bone volume
BIC: Bone–implant contact
BCR: Bone contact ratio
BD: Bone density
MTA: Mineral trioxide aggregate
ALP: Alkaline phosphatase
DSSP: Dentin sialophosphoprotein
DMP1: Dentin matrix acidic phosphoprotein 1
OCN: Osteocalcin
DPSCs: Dental pulp stem cells
a-TCP: a-Tricalcium phosphate
HDPCS: Human dental pulp cells
TNF-α: Tumor necrosis factor-α
Cyr61: Cysteine-rich 61
CCL2: C-C Motif chemokine ligand 2
CMC: Carboxymethyl cellulose
PDL: Periodontal ligament
BMD: Bone mineral density
GI: Gingival index
SMV: Simvastatin
FGG: Free gingival graft
VAS: Visual analogue scale
RSV: Rosuvastatin
IR: Irradiation
miR-21i: MicroRNA-21 inhibitor
SACC-LM: Lung metastatic salivary adenoid cystic carcinoma
HNSCC: Head and neck squamous cell carcinoma
SCC: Squamous cell carcinoma
RhoC: Ras homolog gene family member C
ERK1/2: Extracellular signal-regulated Kinase ½
STAT3: Signal transducer and activator of transcription 3OM: Oral mucositis
5-FU: 5-Xuorouracil
